# *Aedes* mosquito surveillance and the use of a larvicide for vector control in a rural area of the Lao People’s Democratic Republic

**DOI:** 10.1186/s41182-020-00242-7

**Published:** 2020-06-30

**Authors:** Pheophet Lamaningao, Seiji Kanda, Takaki Shimono, Somchit Inthavongsack, Thonelakhanh Xaypangna, Toshimasa Nishiyama

**Affiliations:** 1 Department of Hygiene and Public Health, Kansai Medical University, Hirakata, Osaka, Japan; 2 Regenerative Research Center for Intractable Diseases, Kansai Medical University, Hirakata, Osaka, Japan; 3 Station of Malariology, Parasitology, and Entomology, Khammouane Provincial Health Department, Thakhek, Khammouane Province Lao People’s Democratic Republic; 4 Khammouane Provincial Health Department, Thakhek, Khammouane Province Lao People’s Democratic Republic

**Keywords:** *Aedes aegypti*, *Aedes albopictus*, Dengue, Larvicide, Laos, Lao PDR, Pyriproxyfen, Rural, SumiLarv®2MR

## Abstract

**Background:**

Refillable water containers are commonly used in rural areas of Lao PDR, and they act as *Aedes* mosquito breeding sites. *Aedes aegypti* and *Ae. albopictus* mosquitos are transmission vectors for the dengue virus, which causes dengue fever.

**Methods:**

Two isolated rural villages in the central part of Lao PDR were selected as study sites. In the intervention village, domestic water containers were continuously treated with a long-lasting matrix release formulation, containing pyriproxyfen, named SumiLarv®2MR. In the control village, entomological activity was monitored, but no intervention was performed. Baseline data were collected in both villages during the late rainy season (October 2017) then distributed SumiLarv®2MR disks in intervention village. This data was compared with data collected during the intervention periods in the dry season (February 2018), rainy season (July 2018 and 2019), and late rainy season (September 2018) in the region.

**Results:**

Compared with the baseline data (20.24%), the percentage of water containers infested with *Ae. aegypti* larvae was significantly decreased in the treated village, especially in the rainy seasons in July 2018 (4.11%; *P* < 0.001) and July 2019 (2.46%; *P* < 0.001), while the percentage of water containers infested with *Ae. albopictus* larvae did not decrease significantly in prevalence. No reduction in the frequency of *Aedes* species was seen in the control village. The *Ae. albopictus* liked to breed in small habitats (the median water volume of its habitats was 5 L and 10 L in the control and treated village, respectively, while the equivalent values for *Ae. aegypti* were 30 L and 50 L, respectively).

**Conclusion:**

The treatment of refillable water storage containers in a rural village with SumiLarv®2MR disks led to significant reductions in the *Ae. aegypti* population. However, the *Ae. albopictus* population did not decrease in either the control or treated village. This discrepancy was due to differences in habitat-seeking behaviors and preferred breeding sites such as types of water, water container, and water volume, then led to the differences in results of mosquito prevalence after SumiLarv®2MR disk treatments. The SumiLarv®2MR disk treatment was proven to be effective against the primary dengue-virus vector mosquitoes, *Ae. aegypti*.

## Introduction

Dengue fever (DF) is a mosquito-borne infectious disease, which is prevalent throughout the world; however, it mainly occurs in tropical and subtropical areas, as well as in Southeast Asia, including Lao PDR. DF is caused by dengue virus (DENV) switching in phylogenetic lineages among 4 serotypes (DENV-1, DENV-2, DENV-3, and DENV-4). The transmission of DF in the urban and rural area throughout Lao PDR was reported with scatter province to province in seasonal, especially in rainfall of each year. However, the large outbreaks occur in Lao PDR approximately every 2–5 years. The important recent DF outbreaks with estimated cases of infection were reported by the Ministry of Health, Lao PDR, and the World Health Organization (WHO) [1, 2]. Since 2010, all four serotypes of DENV were associated with the outbreak in the country in 2010, 2013, and 2019, and DENV-2 was the predominant serotype that was involved in the outbreaks.

The DENVs are commonly transmitted by two mosquito vectors, *Aedes aegypti* and *Ae. albopictus*, which also transmit other arboviruses, such as chikungunya virus and Zika virus. They transfer the viruses to humans and can facilitate indirect human-to-human transmission as well, especially in the case of DENV. There is no specific treatment for DF, but medical care provided by experienced physicians and nurses can save lives.

Vector control is the method of choice for preventing DF infections, and the application of appropriate insecticides to outdoor water storage containers has been suggested to be an effective mosquito vector control activity by WHO. However, *Aedes* mosquitos have been reported to exhibit larvicidal resistance to several larvicidal compounds, including temephos (commercial name Abate), which is widely used for vector control because it exhibits residual efficacy for 3 months [3].

As reported by WHO, 80% of the water storage containers in Lao PDR, especially in rural areas, refillable domestic water containers, including jars, drums and concrete tanks, act as mosquito breeding sites, where the insects can lay their eggs [4]. SumiLarv®2MR is a new larvicidal formulation for mosquito vector control, which is slowly secreted into water and has been reported to exhibit long-lasting effectiveness (at least 6 months) when added to refillable water containers. The aim of this study was to examine the efficacy of SumiLarv®2MR, especially against *Aedes* spp. populations in rural villages in Lao PDR.

## Materials and methods

### Study sites

**Fig. 1:**
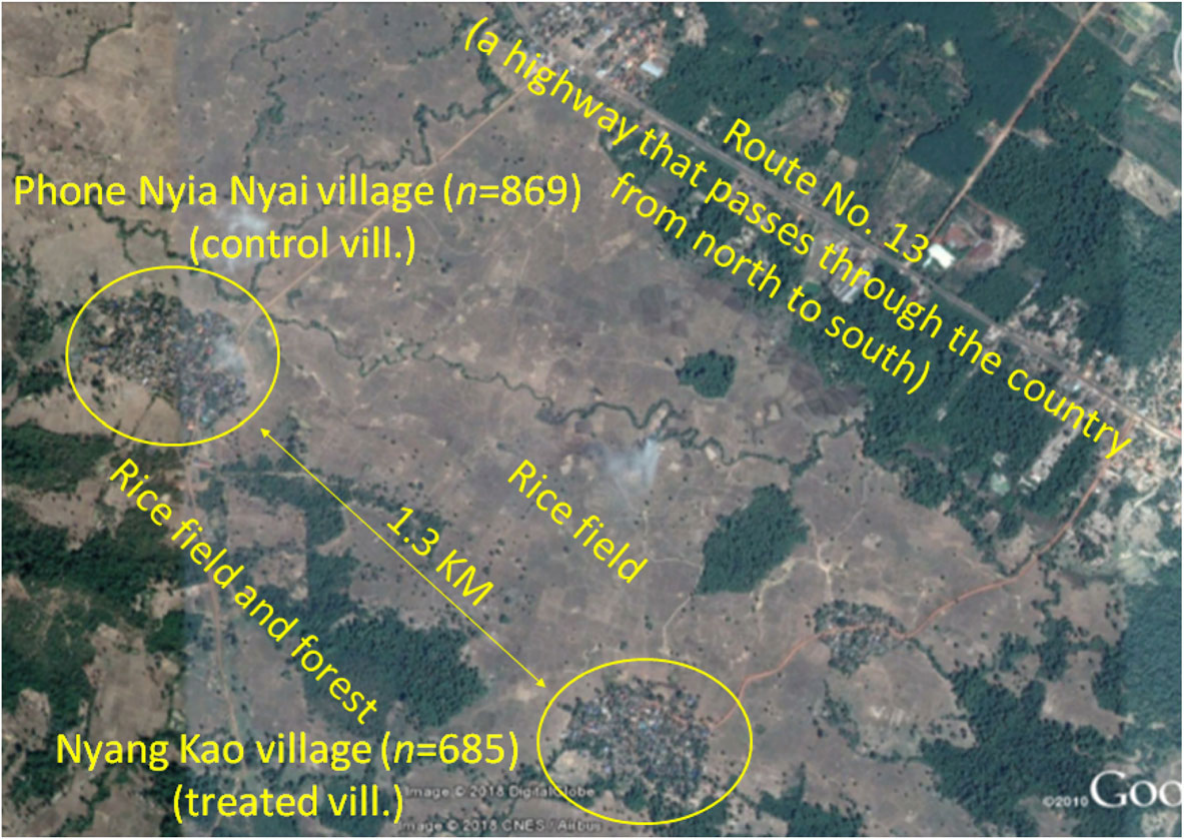
Two villages in Thakhek district, Khammouane Province. Aerial distances were measured using Google Maps

Two similar isolated villages in a rural area is 15 km far from center town of Thakhek District, Khammouane Province, in the central part of Lao PDR, which shares a border with Nakhon Phanom Province, Thailand, were selected as study sites. Phone Nyia Nyai village (17°18′53.69′′N, 104°54′18.63′′E), which included 180 households and had 869 inhabitants in 2015, was selected as the control village, in which entomological activity was conducted, but water containers were not treated with the larvicidal product. On the other hand, Nyang Khao village (17°18′20′′N, 104°55′20.33′′E), which consisted of 143 households and had 685 inhabitants in 2015, was selected as the intervention village, in which refillable domestic water containers as well as suspected mosquito breeding sites were treated with an insecticide (SumiLarv®2MR). These villages are agricultural communities. They are surrounded by paddy fields and are approximately 1.3 km apart from each other, according to aerial distance measurements made using Google Maps (Fig. 1).

### Community preparation and ethics statement

Discussions with the community were held in both villages in October 2017 to explain the details of the research to the local residents and to ensure that the villagers understood the study’s purpose. Villagers and schoolchildren were invited to temples, which functioned as community centers for the villages. All of the activities that were scheduled to be implemented during the study period, such as the entomological monitoring and the distribution of the insecticide in the intervention village, were explained to the villagers. Permission to distribute the larvicidal product in refillable water containers and perform entomological monitoring was received from each household.

### Categorization of containers

The SumiLarv®2MR disks were used as recommended, i.e., one disk was added for each 40 L of volume of the container. Therefore, the containers were categorized into those with volumes of ≤ 40 L and > 40 L. The types of container in the > 40 L group included cement tanks, jars, and plastic drums, whereas the containers in the ≤ 40 L group included buckets, jars, pots, and used tires, as well as small cement tanks and ground pools.

### Insecticide

A long-lasting matrix-release formulation, containing 2% (w/w) pyriproxyfen, SumiLarv®2MR (Sumitomo Chemical Co. Ltd., Tokyo, Japan), was used for the intervention. This formulation exhibits sustained efficacy for at least 6 months [5, 6] and was recommended by the WHO for controlling mosquito larvae [7]. It is recommended that one SumiLarv®2MR disk should be added for each 40 L of water; therefore, more disks were added to water containers with volumes of > 40 L, whereas for water containers with volumes of ≤ 40 L, a disk was cut into equally sized small pieces, and an appropriate number of pieces was added to the container. SumiLarv®2MR disks remain visible after they are added to water storage containers, and they do not affect the smell of the treated water. Since pyriproxyfen is a juvenile hormone analog and acts as an insect growth regulator, it affects the pupal stage by stopping it from turning into an adult mosquito, which contributes to reducing mosquito populations in treated areas. As the disks inhibit the emergence of adult mosquitos from pupae, but do not kill larvae, it is normal to find mosquito larvae in refillable water storage containers that have been treated with SumiLarv®2MR disks.

### Larval survey

In October (the last month of the rainy season in Lao PDR) in 2017, there was a post-seasonal DF outbreak in the region. An entomological survey to obtain the baseline data was first performed by randomly visiting 30 households in the control village (Phone Nyia Nyai village) and 30 households in the intervention village (Nyang Kao village) and inspecting the mosquito larvae populations in different types of water containers as a pre-intervention activity. Then, SumiLarv®2MR disks were added to refillable domestic water containers and other containers that were suspected to be potential mosquito breeding sites in the intervention village. The visits were repeated randomly in the post-intervention period, i.e., in the control and intervention villages 32 and 45 households were visited in February 2018, 32 and 32 households were visited in July 2018, and 41 and 50 households were visited in September 2018, respectively. The last mosquito larvae inspection was conducted in July 2019 (the rainy season) and involved 34 households in the control village and 48 households in the intervention village. All of the mosquito larval surveys were undertaken without warning the community first, and they were completed within 4 to 5 h.

All water containers that could possibly act as breeding grounds for *Aedes* spp., such as jars, plastic drums, buckets, cement tanks, barrel, used tires, and discarded waste, were inspected for the presence of mosquito larvae. Large containers, such as concrete tanks, jars, and plastic drums, were sampled using netting (five times per container). On the other hand, to collect larvae from containers with capacities of < 10 or 20 L, all of the water within the container was poured into a beating net tray. The presence of any stage of *Aedes* or *Culex* instar larvae was recorded for each container. All mosquito larvae were collected, placed in PET bottles (capacity 250 mL), and brought to the laboratory for identification. The larvae were mounted on glass slides and identified using a microscope, according to a previously described method [8]. The inspection of the mosquito larvae was conducted by checking 10 larvae per container in cases in which the container was only infested with *Aedes* spp.; however, 15 larvae were checked in cases in which both *Aedes* and *Culex* spp. were present. *Aedes* and *Culex* spp. were identified using the naked eye before they were mounted onto glass slides. If both *Aedes* and *Culex* spp. were present, then 5 larvae were recorded for *Culex* spp. per container, and 10 larvae *Aedes* spp. were identified to distinguish between *Ae. aegypti* and *Ae. albopictus*. If only one mosquito larva was found in a container, then the container was recorded as a positive container for that mosquito species.

### Treatment in the intervention village

After the baseline entomological survey was completed, all households in Nyang Kao village (the intervention village) were visited, and permission to drop SumiLarv®2MR disks into refillable water storage containers, such as barrels, basins, buckets, cement tanks, jars, and plastic drums, that were in daily use, as well as containers that were not in daily use, but were considered to be suitable targets for treatment, as they were suspected to be breeding sites for *Aedes* mosquitoes, was obtained. All of the households in the village (143 households) agreed to the use of the SumiLarv®2MR disks. Based on the time available for our fieldwork, the SumiLarv®2MR disks were first distributed in October 2017 and then were distributed again in February, July, and September of 2018 and in February 2019, before the last entomological survey was conducted in July 2019. All of the treated containers were sprayed with color to allow them to be tracked.

### Control containers in the intervention village

Bamboo intermodal spacing approximates 30 cm of length and 10 cm of inner diameter, one edge with node and one edge is open, with capacity to hold water of approximately 2 L. Of 16 bamboos used as control container traps, the bamboos were put for households located in center and edge areas of the intervention village during the dry season in February 2019. Two third of bamboo length was put under the ground; 1 L of water was added and treated with one fourth of a SumiLarv®2MR disk. The purpose of using control containers is to inspect mosquito larval species in rainy season in July 2019 after the result of mosquito larval survey showed during rainy season in July 2018 that *Ae. albopictus* species was not decreased likes *Ae. aegypti*.

### Data analysis

The percentage of water-holding containers that were infested with mosquito larvae was calculated for each mosquito species based on the WHO guidelines [9]. The prevalence of presence/absence for mosquito larvae, including SumiLarv®2MR disks in the containers and preferences of water types, was calculated using Fisher’s exact test. The differences in the amount of water (water volume) in the containers that infested with mosquito larvae were calculated using Mann-Whitney *U* test. The statistical analyses were performed using JMP 11.2.1 (SAS Institute Japan Inc., Tokyo, Japan), with *P* values of < 0.05 considered significant.

## Results

### Prevalence of larval infestation

**Fig. 2:**
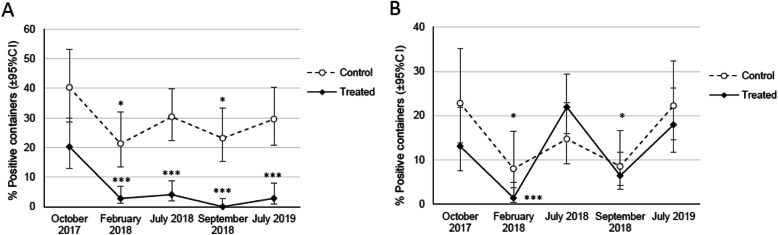
Results of the larval surveys of the control and treated villages conducted during the pre-intervention and intervention periods. **a** The prevalence rate of *Ae. aegypti* species in pre-intervention period (October 2017) in the control village (23/57) and the treated village (17/84), followed by intervention periods, in February 2018 (16/75, 4/143), in July 2018 (31/102, 6/146), in September 2018 (19/82, 0/140), and in July 2019 (24/81, 3/106). **b** The prevalence rate of *Ae. albopictus* species in pre-intervention period (October 2017) in the control village (13/57) and the treated village (11/84), followed by intervention periods, in February 2018 (6/75, 2/143), in July 2018 (15/102, 32/146), in September 2018 (7/82, 9/140), and in July 2019 (18/81, 19/106). *P* values were calculated using Fisher’s exact test (in control village and treated village: pre-intervention in October 2017 vs. intervention periods *P < 0.05 and ***P < 0.001)

The results of the larval surveys of the control and treated villages conducted during the pre-intervention (late rainy season in October 2017) and the intervention periods (dry season in February 2018, rainy season in July 2018, 2019 and late rainy season in September 2018) for changing the prevalence of the two mosquito vectors transmit DENV are shown in Fig. 2.

In Fig. 2a, the difference in percentage of containers infested with *Ae. aegypti* larvae in the treated village was significantly decreased when compared with pre-intervention period (baseline data 20.24%), especially in rainy season of July 2018 (4.11%, *P* < 0.001), July 2019 (2.83%, *P* < 0.001), and high significant difference in September 2018 (late rainy season, *P* < 0.001). On the other hand, the percentage of *Ae. albopictus* was not significantly decreased as *Ae. aegypti* species at the same periods except only in the dry season of February 2018 because it was cool-dry and supposed not available for the increasing population as in the rainy season (Fig. 2b). In the control village, there was no reduction in the frequency of *Aedes* species seen which compared to the pre-intervention period. The prevalence rates were dropped in the dry and late rainy season (Fig. 2a and b). However, the numbers of *Ae. albopictus* was higher in control village than the treated village even in the dry season (in February 2018).

**Fig. 3:**
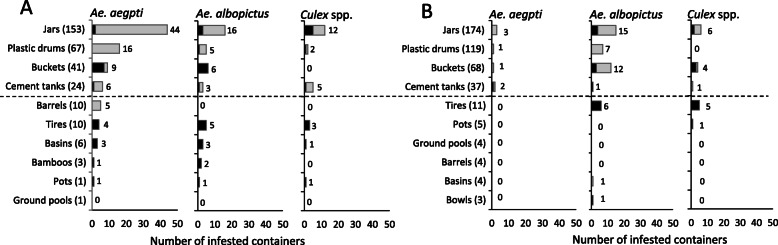
Comparison of accumulated number of containers that were examined and infested with *Ae. aegypti*, *Ae. albopictus*, and *Culex* spp. mosquito larvae and whether the associated water was in daily use or stagnant in two villages. The analyzed data showed the total numbers of infested containers that were obtained in pre-intervention period (October 2017) and intervention periods (February, July, and September 2018) in the control village (**a**) and in the treated village (**b**). The numbers in parenthesis indicate the sample size for each type of containers that were examined, while the numbers without the parenthesis indicate infested number mosquito larvae. The dash line indicated division as the upper were 4 main container types that commonly used in the village and were habitats for mosquito as the analysis result was described in the “Materials and methods” section. The grey bar indicated water in daily use, and the black bar indicated stagnant water

In over all, the water containers such as bamboos, barrels, basins, bowls, buckets, cement tanks, ground pools, jars, plastic drums, pots, and used tires which were infested by the mosquito larvae in both villages were investigated during the pre-intervention period and the intervention periods (Fig. 3). The containers in which mosquito larvae most commonly habited were four main container types (jars, plastic drums, buckets, and concrete tanks). These containers were also commonly used as the domestic refillable water containers in these villages. The difference in the percentage of prevalence in the four main container types also was calculated using accumulated data during the intervention periods (February, July, and September in 2018) for treated village, while in control village included baseline data in October 2017. In the control village, those containers were infested with *Ae. aegypti* 26.31% (75/285) and with *Ae. albopictus* 10.52% (30/285). In treated village, it was infested with *Ae. aegypti* 1.76% (7/398) and with *Ae. albopictus* 8.79% (35/398). By results, the numbers of *Ae. aegypti* larval population also decreased in number of infestation in treated villages, while the *Ae. albopictus* larval population were similar in both villages.

### Water condition preferences

**Fig. 4:**
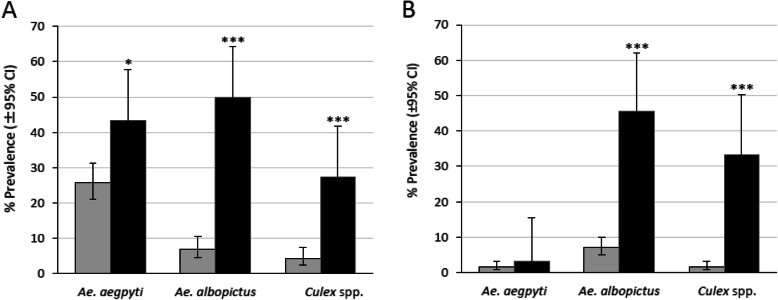
Differences in the preference for water types among mosquito larvae species in two villages. **a** Prevalence rate in the control village for water in daily use (number of infestation by *Ae. aegypti*, *Ae. albopictus*, and *Culex* spp., respectively 70, 19, 12; *n* = 272) and stagnant water (19, 22, 12; *n* = 44) that was obtained in the pre-intervention period (October 2017) and intervention periods (February, July, and September 2018) were used to analyze. **b** Prevalence rate in the treated village for water in daily use (6, 28, 6; *n* = 396) and stagnant water (1, 15, 11; *n* = 33) obtained during intervention periods (February, July, and September 2018) was used to analyze. The comparison showed that *Ae. albopictus* strongly preferred stagnant water, while *Ae. aegypti* did not prefer stagnant water as strongly as *Ae. albopictus. P* values were calculated using Fisher’s exact test (water in daily use vs. stagnant water: *P < 0.05, ***P < 0.001). The grey columns indicated water in daily use and the black columns indicated stagnant water

According to the examination of the water condition preferences of each mosquito species, i.e., whether they preferred water that was in daily use or stagnant water. The number of containers infested with the mosquito larvae (*Ae. aegypti*, *Ae. albopictus*, and *Culex* spp.) showed in both villages that *Ae. albopictus* seemed to prefer the stagnant water more than the water in daily use with the prevalence rates 7.00% (water in daily use) and 50% (stagnant water) in the control village, while in the treated village 7.10% and 45.50%, respectively with significant difference of *P* value at *P* < 0.001 (Fig. 4a, b).

### The relationship of the water volumes and mosquito larval habitats

**Fig. 5:**
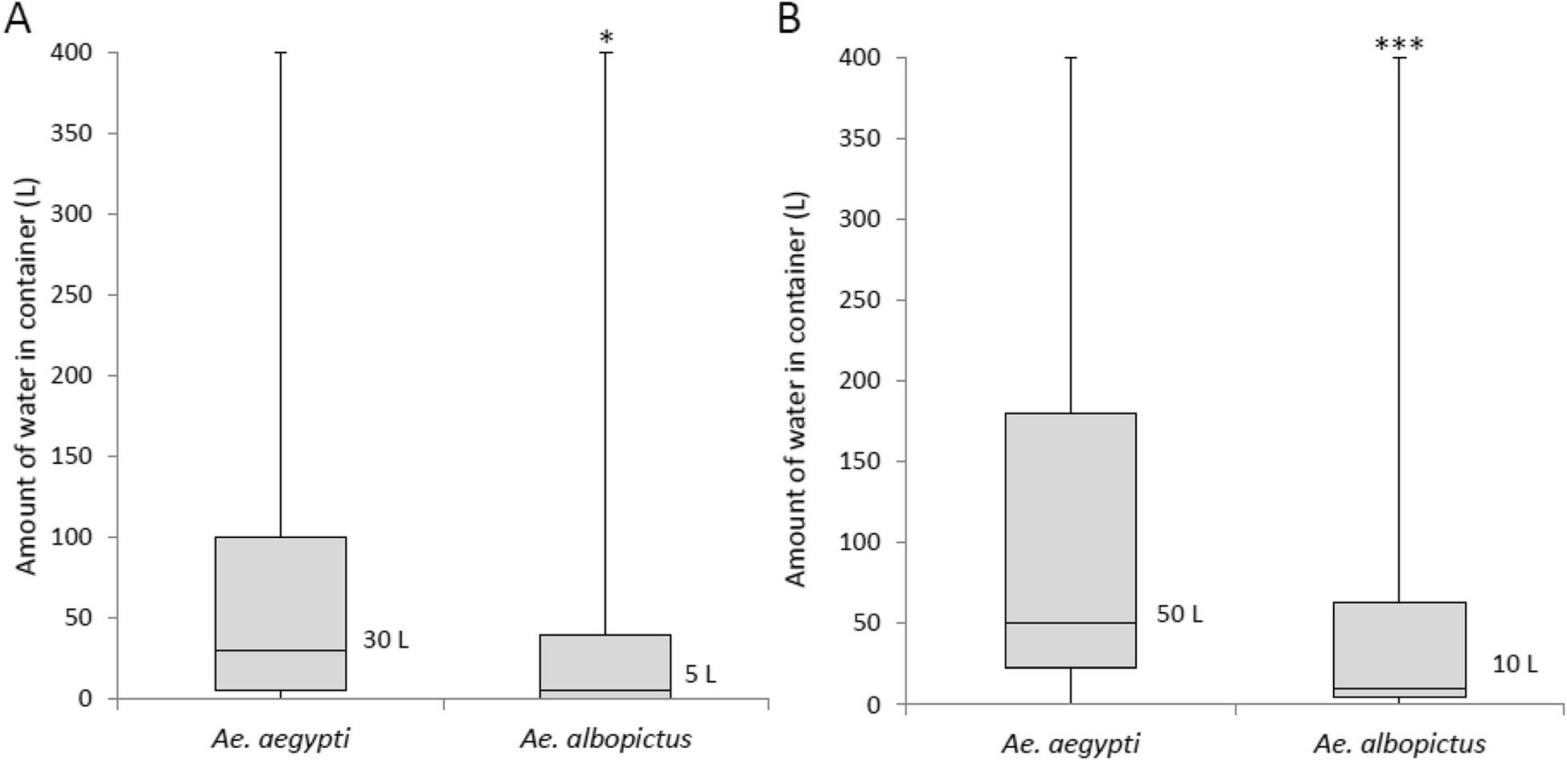
Differences in the amounts of water in the containers infested with mosquito larvae. **a** Data of control village, *Ae. aegypti* (*n* = 89) and *Ae. albopictus* (*n* = 41); **b** Data of treated village, *Ae. aegypti* (*n* = 24) and *Ae. albopictus* (*n* = 54). The analyzed data were obtained in the pre-intervention period (October 2017) and intervention periods (February, July, and September 2018). Horizontal line within box indicates median value; upper and lower borders of the boxes indicate the 75th and 25th percentile, respectively; and vertical line represents minimum and the maximum values. *P* values were calculated using the Mann-Whitney *U* test. The comparison between water volume of containers infested by *Aedes* spp. with significant difference (*Ae. aegypti* vs. *Ae. albopictus*: *P < 0.05, ***P < 0.001)

A comparison between the water volumes of the containers infested by the two *Aedes* spp. in the control village (Fig. 5a) revealed a significant difference (*P* < 0.05). Immature *Ae. aegypti* infested containers with a wide range of water volumes (median 30 L, range 0.2–400 L), whereas immature *Ae. albopictus* tended to inhabit small water containers (median 5 L, range 0.2–400 L). The water volumes of the containers infested by the two *Aedes* spp. also differed significantly (*P* < 0.001) in the treated village (Fig. 5b). Immature *Ae. aegypti* infested containers with a wide range of water volumes (median 50 L, range 0.5–400 L), whereas immature *Ae. albopictus* tended to inhabit small water containers (median 10 L, range 0.1–400 L).

### Presence of disks and mosquito larval relationship in the intervention village

**Fig. 6:**
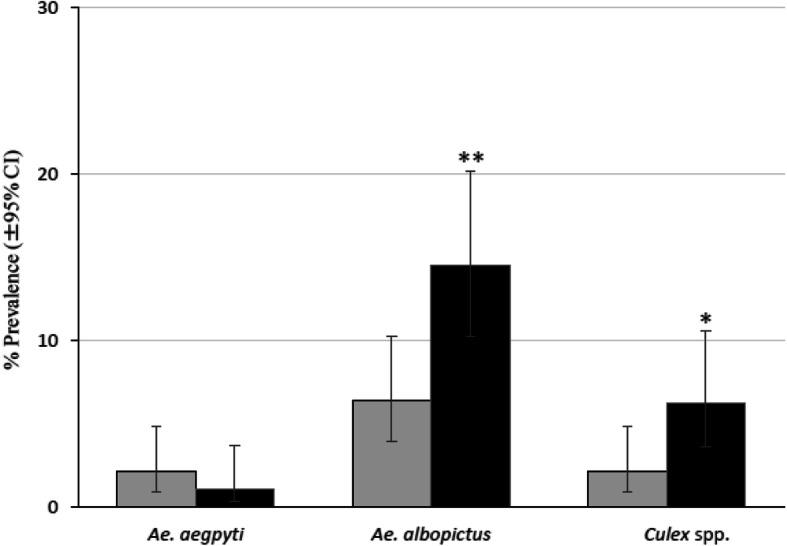
Relationship between the prevalence of larval species (*Ae. aegypti*, *Ae. albopictus*, and *Culex* spp.) and the presence/absence of the SumiLarv®2MR disks in the treated village. The containers that contained SumiLarv®2MR disks (number of infestation by *Ae. aegypti*, *Ae. albopictus*, and *Culex* spp., respectively 5, 15, 5; *n* = 236) and did not contain SumiLarv®2MR disks (2, 28, 12; *n* = 193) that were obtained in the intervention periods (February, July, and September 2018) were used to analyze. *P* values were calculated using Fisher’s exact test (SumiLarv®2MR disk present vs. SumiLarv®2MR disk absent: *P < 0.05, **P < 0.01). The grey columns indicated SumiLarv®2MR disk present and the black columns SumiLarv®2MR disk absent

An analysis of the relationship between the prevalence of larval species and the presence/absence of SumiLarv®2MR disks revealed that the difference of *Ae. aegypti* prevalence was not significant, while the prevalence rates for *Ae. albopictus* was significantly higher in the habitats that did not contain SumiLarv®2MR disks (*P* < 0.01, Fig. 6).

The tracking of the SumiLarv®2MR disks was performed after 10 months of treatment (October 2017 to July 2018). We monitored the containers by conducting inspections of each container that had been sprayed with color. Out of a total of 108 containers in 32 households, 72.23% still contained SumiLarv®2MR disks. Among the containers with volumes of ≤ 40 L, the percentage of containers that contained SumiLarv®2MR disks was 48% (*n* = 25) for buckets or basins and 63% (*n* = 27) for jars. As for the containers with volumes of > 40 L, the percentage of containers that contained SumiLarv®2MR disks was 80% (*n* = 30) for plastic drums, 93.75 % (*n* = 16) for jars, and 100% (*n* = 10) for cement tanks.

### Control containers in intervention village

In terms of the control containers which used bamboo as traps in the intervention village in July 2019 found out only *Ae. albopictus* infested was 93.75% (*n* = 16). This result showed that there were more other habitats for *Ae. albopictus* more than domestic-refilled water containers in the village.

## Discussion

The entomological survey results obtained in this study suggested that treating water containers in a rural village in Thakhek District, Lao PDR, with SumiLarv®2MR disks led to a reduction in the population density of *Ae. aegypti*. However, the prevalence of *Ae. albopictus* did not differ significantly between the control and treated villages. This discrepancy was probably caused by differences in breeding behavior between the two *Aedes* mosquito species. Small containers, such as discarded tires, unused jars, and bamboo, containing small amounts of stagnant water were preferred by *Ae. albopictus*, and these containers could not be treated as effectively with SumiLarv®2MR disks. These habitats could allow *Ae. albopictus* to continually rebreed. A previous study conducted in Thailand reported that *Ae. aegypti* prefers to breed in water storage jars, whereas *Ae. albopictus* inhabits various water sources, such as discarded cans and used tires [10]. In this study, we tried to check this phenomenon to use the bamboo traps which contain small volume of water as the control containers in treated village, and all the bamboo traps were infested by only *Ae. albopictus*. The studies carried out in Lao PDR also demonstrated that *Ae. albopictus* was found more frequently in small water containers and discarded waste than in large storage containers [11–13]. In the present study, we found that the two examined *Aedes* species prefer different water volumes. The median volume of the water infested with *Ae. albopictus* was 5 L in the control village and 10 L in the treated village, which were significantly smaller than the median volumes of the water infested with *Ae. aegypti* (30 L in the control village and 50 L in the treated village) (Fig. 5). Furthermore, *Ae. albopictus* and *Culex* spp. seemed to strongly prefer stagnant water over water that was in daily use, while *Ae. aegypti* preferred stagnant water less than *Ae. albopictus* (Fig. 4). Moreover, most of the containers in which *Ae. albopictus* were found did not contain SumiLarv®2MR disks, and most of them contained small volumes of stagnant water (Fig. 6).

Nevertheless, in February and September 2018 the prevalence rates of both species of *Aedes* larvae were significantly reduced in both villages. This finding might have been related to the timing of the dry and late rainy seasons in the region, i.e., there was few rainfalls, and hence, there would have been a lack of mosquito breeding sites in February and September. On the other hand, the population of *Aedes* mosquitos continuously increased in the control village, while in the treated village only the prevalence of *Ae. albopictus* increased in rainy season in July (Fig. 2b). July is the middle of the rainy season, and the rainfalls provide places for mosquitos to lay eggs and for larval development, and the temperature at this time usually facilitates *Aedes* population growth [14, 15].

In this study, there was no language barrier between the researchers and the inhabitants of either village. All of the villagers belonged to a Lao minority group. In Lao PDR, there are 49 official ethnic groups, and different ethnic groups have different dialects, customs, and beliefs, including regarding health-seeking behavior. Therefore, there are many challenges with getting a community to participate in any activity that is carried out at the village level. The main challenge is the need for all community members to understand the steps or processes required to create equality during the activities. We recognized that community members differ, e.g., some people have educational qualifications, while others do not. In our study, the water storage containers that were treated with SumiLarv®2MR disks were tracked with colored spray so that we could monitor the presence/absence of the disks and the container usage of the villagers. Based on our findings, we suggest that the behavior of villagers had a very important impact because according to the aims of the study SumiLarv®2MR disks should have been present in all of the treated containers; however, disks were no longer present in many of the containers with volumes of ≤ 40 L, such as buckets and basins, while most of the containers with volumes of >40 L, such as plastic drums and jars, and all of the cement tanks contained SumiLarv®2MR disks. This might have been due to the fact that the small containers were light, which made them easy to move around the house, e.g., to use them for other purposes, and easy to wash, and the villagers might have then forgotten to put the disks back. In contrast, the large containers were heavy and were not easy to move.

### Strengths and limitations

This study is a pilot of a field trial study in a rural area in a south-central part of Lao PDR. The villages in this area were isolated to each other, which is a typical settlement of villages in many other rural areas in Lao PDR, it was an appropriate environment for doing a trial study such this time. However, this study still remained some points that have to be considered as a limitation of the study: (1) small sample size of only 2 villages included for studying, a village as control and a village as intervention, (2) village selection were differences of baseline data mosquito larval prevalence between control and intervention villages was different for a half of prevalence rate. Therefore, to have a clear picture on which this study was found, it requires further studies by scaling up the number of villages.

## Conclusions

This field study examined the use of SumiLarv®2MR disks to treat water storage containers. The matrix-release formulation of these disks has been shown to maintain effective concentrations of the active ingredients in treated water for at least 6 months in Malaysia [5] and Japan [6]. Treating water storage containers in rural areas using SumiLarv®2MR would reduce the number of treatments required per year, which would significantly reduce the operational costs of anti-mosquito programs. In the current study, the treatment led to a reduction in the *Ae. aegypti* population in all seasons in the treated village. However, the number of *Ae. albopictus* did not decrease, especially in the rainy season. This happened because the two *Aedes* mosquito species prefer different breeding habitats, e.g., in terms of the container type, water type, and water volume, rather than due to problems with the residual efficacy of SumiLarv®2MR. Therefore, controlling arbovirus transmission vectors, especially *Ae. albopictus*, in rural areas requires further activities, such as environmental management of the community, in addition to the use of SumiLarv®2MR disks [16]. Moreover, the findings of this study will encourage health authorities to consider more policies/strategies that aim to increase surveillance and control of arbovirus disease vectors, e.g., by using an appropriate insecticide in tandem with environmental management of the community to achieve mosquito vector control.

## Data Availability

Not applicable.

## Abbreviations

Lao PDR: Lao People’s Democratic Republic
*Ae.*: *Aedes*
DF: Dengue fever
DENV: Dengue virus
WHO: World Health Organization
*Culex* spp.: *Culex* species pluralis
v.s.: Versus
NECHR: National Ethics Committee for Health Research
